# Malaria elimination in Lao PDR: the challenges associated with population mobility

**DOI:** 10.1186/s40249-017-0283-5

**Published:** 2017-04-25

**Authors:** Sengchanh Kounnavong, Deyer Gopinath, Bouasy Hongvanthong, Chanthalone Khamkong, Odai Sichanthongthip

**Affiliations:** 1 National Institute of Public Health, Ministry of Health, Ban Kaognot, Samsenthai Road, Sisattanak District, Vientiane, Lao PDR; 2 World Health Organization (WHO), Country Office, Nonthaburi, Thailand; 3 Center for Malaria, Parasitology, and Entomology (CMPE), Ministry of Health, Vientiane, Lao PDR

**Keywords:** Malaria, Malaria control program, Malaria elimination, Malaria outbreak, Migrants, Mobile populations, Lao PDR

## Abstract

Although the Lao People’s Democratic Republic (Lao PDR) is comparatively small landlocked country with patterns of both in- and out-migration, its human migration situation has been poorly studied. This is despite all of the country’s 18 provinces sharing both official and unofficial border checkpoints with neighboring countries. Economic reforms in the last decade have seen a gradual increase in the promotion of foreign investment, and main towns and transportation networks have been expanding thus offering new opportunities for livelihoods and economic activities.

In the last decade, there has also been a significant reduction of reported malaria cases in Lao PDR and while this is an important prerequisite for eliminating malaria in the country, malaria outbreaks reported in the last four years suggest that population mobility, particularly in the south, is an important factor challenging current control efforts.

Bolder investment in social sector spending should be geared towards improving health service provision and utilization, ensuring equitable access to primary health care (including malaria) through efforts to achieve universal health coverage targets. This should be extended to populations that are mobile and migrants. The local government plays a critical role in supporting policy and enforcement issues related to private sector project development in the provinces. Cross-border initiatives with neighboring countries, especially in terms of data sharing, surveillance, and response, is essential. Mechanisms to engage the private sector, especially the informal private sector, needs to be explored within the context of existing regulations and laws. Existing and new interventions for outdoor transmission of malaria, especially in forest settings, for high-risk groups including short- and long-term forest workers and their families, mobile and migrant populations, as well as the military must be combined into integrated packages with innovative delivery mechanisms through social marketing approaches. This should happen at multiple points in the mobility pathway and involve the private sector rather than being fully reliant on the national malaria vertical program

This article based on the review of existing literature from abstracts and full texts, includes published, peer-reviewed English language literature sourced through PubMed and grey literature sources through Google and Google Scholar. The review included also case reports, sector reports, conference proceedings, research reports, epidemiology studies, qualitative studies, and census reports in both Lao and English languages. The authors used the search terms: malaria and mobile populations, malaria control program and elimination, health system performance, malaria outbreak, Lao PDR; and included articles published until June 2015.

**Electronic supplementary material:**

The online version of this article (doi:10.1186/s40249-017-0283-5) contains supplementary material, which is available to authorized users.

## Multilingual abstract

Please see Additional file 1 for translation of the abstract into the five official working languages of the United Nations.

## Background

**Fig. 1:**
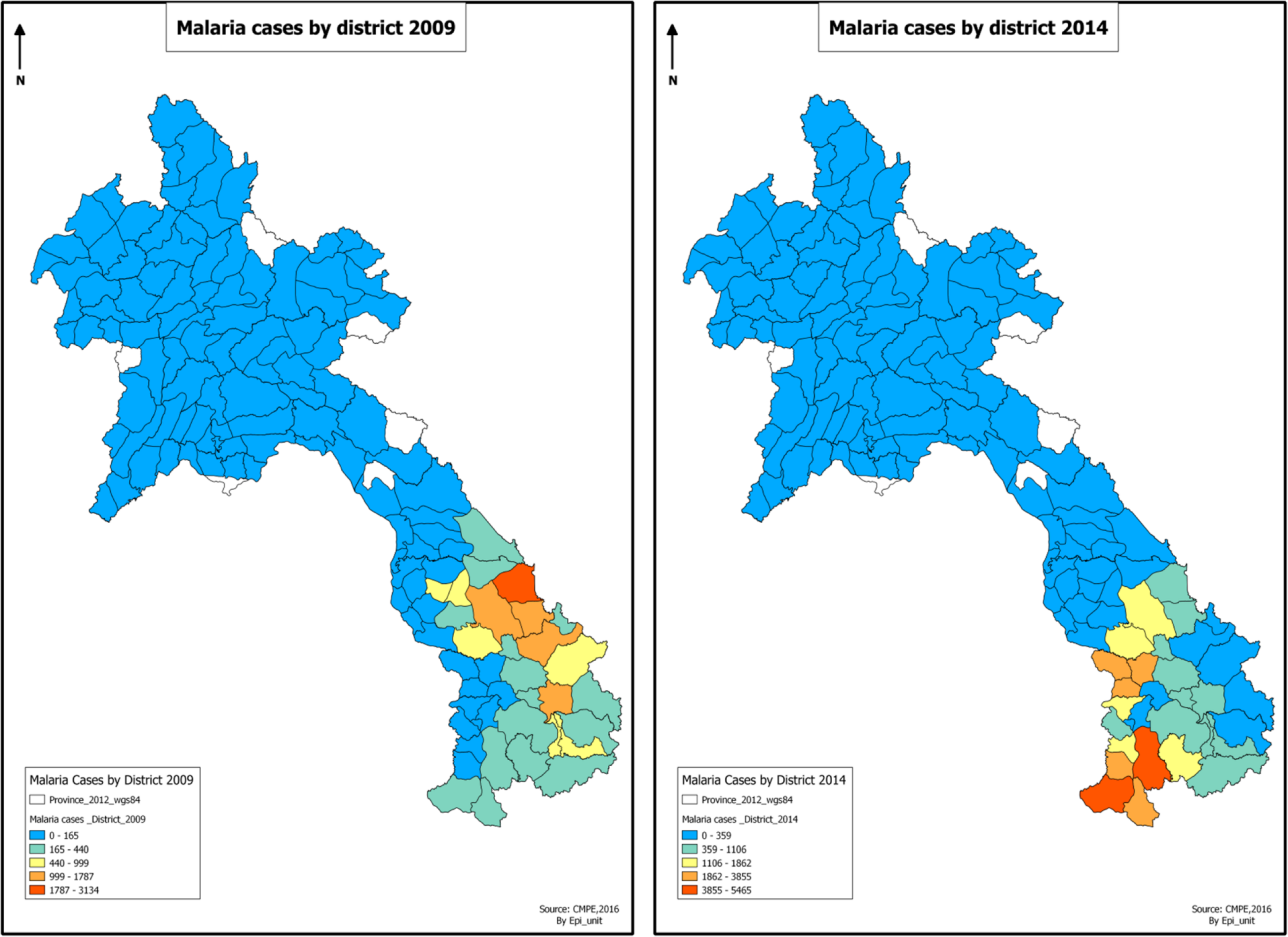
Lao Peoples Democratic Republic: Confirmed malaria cases by district in 2009 and 2014. Confirmed malaria cases in 2009 and 2014 in Lao PDR, by district

Malaria transmission varies across the different ecological zones of Lao People’s Democratic Republic (Lao PDR). The majority of high transmission areas are hilly, forested areas in the southern part of the country where the majority of people are employed in forest-related occupations [1]. Lower rates of transmission are recorded in the plains along the Mekong River and in high altitude areas. Between 2002 and 2012, the number of suspected (showing clinical symptoms) and confirmed malaria cases, and the number of malaria-related deaths fell by 46% (from 85 192 to 46 153) and 77% (from 195 to 44), respectively [2]. However, these gains have been threatened by a large-scale resurgence in the southern half of the country beginning in 2011, with more than 50 000 confirmed cases reported nationally in 2014 [2]. The national annual parasite incidence (API) in 2011 was 2.66 per 1 000 population, but rose to 7.3 in 2014, with the subnational API in the five southern provinces reaching 20.3 per 1 000 [2]. This resurgence was focused in Savannakhet, Saravan, Sekong, Attapeu, and Champasak, collectively accounting for 96% of the total cases in 2014. However, over the same period, the northern half of the country has made rapid progress towards subnational elimination goals, recording 708 cases (API of 0.21) in 2014. Out of a total population of almost seven million, approximately 36% live in high transmission areas (API >1 per 1 000), 23% live in low transmission areas (API < 1), and the remaining 41% live in malaria-free areas [ibid] (see Fig. 1).

### Malaria in mobile and migrant populations (MMPs)

**Fig. 2:**
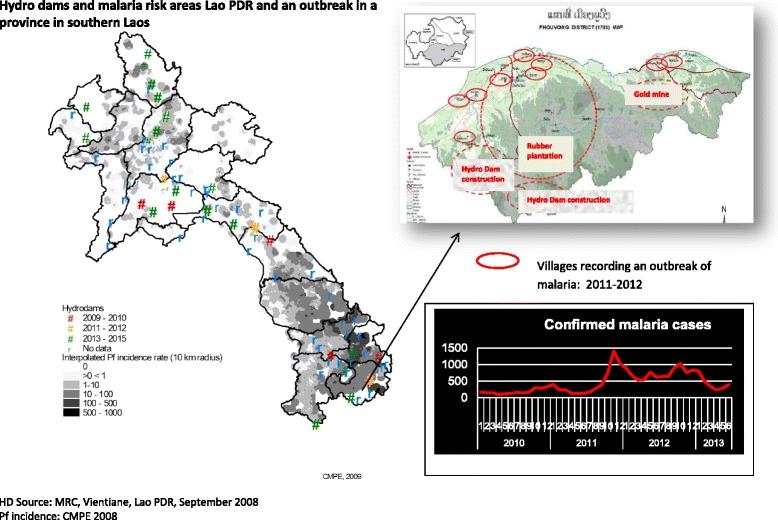
Hydro dams and malaria risk areas in Lao PDR: an outbreak in a province in southern Laos

Population groups at risk of acquiring malaria in endemic areas of Lao PDR include both static and mobile populations [2]. The malaria outbreaks in the last five years have been attributed to a number of factors including unregulated deforestation, deforestation related to large-scale development projects (the construction of hydropower dam, roads, mining, plantations, etc.), migration of workers from non-endemic areas, and possibly climatic conditions (rainfall patterns, temperature, and humidity) [3, 4]. However, it is believed that large-scale population movements (both within Lao and across national borders) as well as forest-related economic activities have primarily driven this resurgence of malaria; the vast majority of reported cases (86% in 2014) were adult males. Migrant workers (both registered and unregistered) from neighboring countries employed on development projects in Lao PDR are also at high risk of acquiring malaria, but cases from these groups until 2015 were not routinely captured through the malaria information system (MIS). An assessment of a malaria outbreak in Attapeu in 2011 showed that migrant workers, both from other provinces in Lao PDR and neighboring countries, accounted for approximately 70% of confirmed malaria cases [Deyer G. Brief report on malaria outbreak in the Southern part of Lao PDR. December 2012. Unpublished reports for CMPE and WHO, 3]. The hydropower dam projects in Attapeu involved an estimated 4 000–5 000 workers at the peak of the construction phase in 2012, the majority of whom were Vietnamese and Chinese nationals [CMPE. Malaria Outbreak in Attapeu Province: November - December 2011, Investigation Report, unpublished. 2011] (see Fig. 2). Although control measures were taken, the number of cases did not drop (as of late 2014) to the seasonal low levels seen prior to 2011. However, preliminary findings of a survey conducted by Health Poverty Action [5] in 2015 show that of the 186 mobile and migrant workers interviewed in parts of southern Lao PDR, 66% were Lao while 31% were Vietnamese and the rest were from China, Cambodia, and Thailand, the majority (70%) of whom were adult males. In this group, 85% (158/186) had a blood test of whom 71% tested positive for malaria. In the northern and central provinces, focal outbreaks over 2011-2015 revealed, through outbreak investigations, that causation of the outbreaks was mostly due to resident mobile workers in the northern provinces with relatively low immunity returning from the endemic southern provinces of Attapeu, Champasack, Sekong and Saravanh [4].

### Economic development and changing land use

Lao PDR’s economy grew to 8.1% in 2013 resulting in the World Bank declaring that the country has a fast-growing economy. There has been an average 7% gross domestic product growth over the last two decades, the highest growth rate in Southeast Asia. The country’s population is projected to increase by 27% from 6.4 million in 2010 to 8.8 million in 2030, and it is predicted to achieve its long-term goal of graduating from its ‘least developed country’ status by 2020 [6]. The major contributors to this growth have been the service, mining, hydropower, construction, and food processing industries [ibid], with plans for further expansion in these sectors. As of 2014, a total of 53 hydropower dam projects (signed memorandum of understandings and project development agreements) have been initiated, with 30 of these in the south [7] where malaria is prevalent. In the fiscal year of 2013–2014, the mining industry earned 12.56 trillion LAK (1.56 billion USD) from exports, with the Ministry of Energy and Mining granting licenses to 69 companies operating projects on a total area of 274 663 ha [8]. A recent study showed that almost one third of all land concessions and leases granted involve land categorized as forests. In addition, 23% of all land under investment is categorized as protection forest [9]. While it is currently estimated that 7 out of 10 workers in Lao PDR are engaged in agriculture, it is predicted that 96 000 young people will be looking for jobs annually in the coming decades, which would greatly influence population mobility both across rural and urban areas, and across borders [10].

### Human migration patterns

Rural infrastructure development may bring non-immune people into endemic areas [11], and increase mobility among local surrounding populations and among non-mobile populations in villages to which migrants periodically return [2]. The Asian Highway and Greater Mekong Subregion (GMS) economic corridors that cut through endemic areas in GMS countries and extensively in Lao PDR [12, 13] will mean that an increasing number of people and possibly malaria parasites will move between localities with different malaria endemicity and risks of malaria transmission [14]. In addition to planned development activities, unregulated economic activities such as logging related to constructions of dams particularly in the southern provinces pose challenges for vector control and protection measures, and for prompt detection and treatment of infected cases. Many people when febrile or sick also prefer to return to their home provinces thus increasing the possibility of severe malaria complications and possible death due to delayed treatment [4].

Although all 18 provinces of Lao PDR are bordered with GMS countries, the out-migration patterns are not significantly higher near the borders highlighting that geographical proximity of other countries does not seem to be the most crucial factor stimulating out-bound migration [15]. The high in-migration rates in the more remote districts have been difficult to interpret [16], but are currently known to be driven by large-scale development projects and forest exploitation economies. The rapid rural development in Lao PDR is also attracting people from China, Vietnam, and Cambodia [17, 18], the majority of whom are employed by large private companies, live in unauthorized housing developments, and work as seasonal agricultural laborers or as informal forest workers. Many are unregistered or illegal, thus they try to avoid contact with authorities of any kind and are therefore reluctant to seek health care [2]. It is estimated that there are from 54 135 [17] to 200 000 [19] foreign workers, which is significant given a population of approximately seven million in 2013. The establishment of special economic zones and construction of Chinese markets in major towns suggest that the number of Vietnamese and Chinese migrants could be much higher than the reported figures [20]. Recent anecdotal reports from the Cambodia and Lao PDR border areas reveal that there is more active movement across both land (Dom Kralor-Voeung Kam) and riverine (Koh Chheuteal Thom) border crossing points.

## The current situation: challenges for the health system and strategies to addressing MMPs

### Health care delivery in the community

The main network for health care provision remains the public system with government owned and managed provincial and district hospitals and health centers/clinics. At the village level, there are large numbers of village health volunteers (VHVs), members of community health committees, and traditional birth attendants (TBAs). Members of community health committees and village (zonal) committees are characterized by various health center management arrangements, health promotion activities, and communicable disease prevention campaigns, and are usually linked with staff from their local health centers.

In the period of 2009–2010, there were 53 676 local community members involved in health activities, of whom 14 812 were VHVs, 6 128 were TBAs, 1 222 were traditional healers, and 31 514 were members of village health committees [21]. Since 2013, village malaria volunteers and village malaria workers (paid incentives in malaria endemic villages) have played a key role in the distribution of long-lasting insecticide-treated nets (LLINs), provision of early diagnosis and treatment (EDAT) through the use of rapid diagnostic tests and effective artemisinin-based combination therapy, and reporting of results to the nearest health center on a monthly basis in return for fresh supplies [22]. A scheme currently being initiated in the south expands the VHV and Village Health Worker community case management strategy to a specific focus on mobile populations (plantation workers, seasonal agricultural laborers, forest workers, etc.) [4] involved in all long-term and short-term development projects.

### Malaria surveillance and response

In 2012, malaria reporting forms were redesigned to simplify the process and improve the MIS. This resulted in some enhancement in the accuracy and timeliness of reporting due to the introduction of a cloud-based reporting system [4]. In 2015, MIS forms were revised to routinely disaggregate malaria patients’ place of origin and travel history. However, the current approaches may still fail to capture malaria surveillance data on MMPs for a number of reasons, including, language barriers, legality status of work activities or residence, access to health facilities, capacity of health staff in data management, reporting, and use of information and technology (ICT) tools [23]. The Health Sector Reform Framework of the Ministry of Health aims to establish, as a key element, the District Health Information Software (DHIS2) as an ICT platform to strengthen health information systems across Lao PDR [4] with an integrated malaria, TB, and HIV program module that will require an optimum set of minimal essential data reporting to a set of indicators for each of the diseases [24]. While this effort is a positive achievement for health information systems in the country, in the context of malaria elimination efforts however, the capacity of individual case reporting at the health facility level and the conduct of timely case investigation as part of pre‐elimination and elimination activities needs to be strengthened [25]. An added challenge is the need for relevant data on labor trends and development project databases from non-health sectors, i.e. Ministry of Natural Resources and Environment, Labor, and Energy/Mines, to be included and periodically updated in stratification of malaria risk areas. In addition, there needs to be adequate capacity to analyze these data to forecast health (malaria) trends and incorporate them into program strategy and implementation, as well as funding flexibility to respond to changes in disease trends [14].

### Case management and malaria drug resistance

In Champasak, southern Lao PDR, the therapeutic efficacy of artemether/lumefantrine (Coartem®, the first-line treatment regimen) has been unaffected and cure rates have remained high since 2005. However, a trial conducted in two of the province’s districts in 2013 reported that 22.2% of the patients treated with artemether/lumefantrine were still parasitemic on day 3 after treatment, with the presence of K13 mutations in the circulating parasite population. This confirmed the emergence of artemisinin resistance in southern Lao PDR [26]. These results come from therapeutic efficacy studies (TESs), where patients who are enrolled are those that can be followed up serially over the course of treatment and beyond for blood tests. The proportion of patients with delayed parasite clearance times are believed to be similar or even higher among the highly mobile populations who are excluded from the inclusion criteria of TES.

*Plasmodium vivax* currently accounts for almost 50% of the malaria cases in southern Lao PDR and ongoing pilot studies in three sites to inform a nationwide introduction of primaquine is expected to be a core strategy to prevent the relapse of *P. vivax* infections while also acting as a radical cure for *P. falciparum*. If scaled up nationally, this could be the single most important factor for reducing transmission among returning mobile workers from the south, many of whom have repeated infections in a given year [CMPE, Summary report of the Inception Cross Border meeting for ICC2 Project, Champasak Province, Lao PDR 18–19 August 2016].

### Partnerships with the private sector

After a 2005 drug use survey [27] revealed that 33% (172/521) of patients sought treatment from the private sector (defined as a private pharmacy, clinic or practitioner, drug seller, or mobile drug vendor), the national malaria program initiated a public-private mix (PPM) engagement with registered private pharmacies and clinics in September 2008. Initially, 141 private pharmacies and 9 registered clinics in four malaria endemic provinces were enrolled and trained to provide malaria diagnosis, treatment, and referral, with monthly reports to and periodic supervision from District Malaria Stations.. Following an evaluation, this initiative was geographically scaled up to a total of eight provinces, four of which are in the south of the country. Analysis of the data collected from January 2009 through to February 2013 suggests that the PPM project has contributed to about 7% of the total individuals who were tested in the eight provinces [4]. Micro-planning workshops during the malaria outbreaks in 2012 and 2013 identified and targeted an additional 149 pharmacies and drug vendor sites as a strategy to improve access to malaria services for high-risk mobile population in southern Lao PDR. Although the reporting forms used at these sites capture patients’ place of origin, current reporting and routine surveillance forms and systems are unable, at higher levels of aggregation (provincial and central levels) to disaggregate patients’ place of origin for analysis. However, the selection criteria of PPM sites are based partly on the preferred mode of access by MMPs in these areas [CMPE, unpublished, Evaluation of the PPM initiative in four PPM pilot provinces, October 2009]. There are immediate plans to expand the current PPM initiative to cover all registered private sector providers by 2018 [4].

### Vector control and personal protection among MMPs

While notable attempts have been made since 2012 by the national program to address coverage of MMPs with LLINs, long-lasting insecticide-treated hammocks (LLIHs), and mosquito repellents, high-risk groups that do not have fixed addresses, families that are newly settled in a village, and forest goers in areas of new settlements are missed during the formal mass distribution. A recent survey in the south of Lao PDR, albeit limited in its sampling, revealed that 82% of MMPs used conventional and hammock untreated nets purchased from the market, while only 16% used LLINs distributed free of charge as part of the program. In addition, 26% preferred using mosquito coils, lotion, or spray repellents [5]. This suggests that user preferences and acceptance need to be taken into account when designing appropriate strategies for mobile populations and forest goers. Whilst there is currently a lack of evidence as to the effectiveness of LLIHs and repellents for forest goers in Lao PDR, it is nevertheless a current strategy used for mobile populations in targeted villages, along with raising awareness on the use of protective clothing and repellents when spending nights outdoors. In the meantime, malaria posts and malaria mobile teams that are planned to be established in hotspot areas along the Thailand, Cambodian, and Vietnamese borders will be stocked with Icon Max® (a sachet Kit for long-lasting treatment of mosquito nets) for impregnation of self-purchased untreated net used by forest goers [4].

### The military

The Laotian military has yet to be fully engaged in terms of malaria prevention and case management despite the fact that it is a high‐risk mobile population. Whilst positive attempts have been made to collaborate and coordinate with the Ministry of Defense (MoD) by the Center for Malariology, Parasitology, and Entomology (CMPE), a systematic programmatic and integrated approach for incorporating the military malaria control program into the national strategy is lacking. Efforts have been made to provide army doctors and medics with up‐to‐date training covering the current national guidelines, case management, prevention strategies, and the provision of insecticides. There is now also mutual interest to explore options around insecticide-treated uniforms and repellents, and establish a memorandum of agreement between MoH and MoD to address the problem of malaria among soldiers, including the sharing of information on malaria morbidity and mortality [4].

### Human resources

Health service provision is constrained by serious weaknesses relating to human resources for health. Key issues include limited numbers of qualified health workers, inappropriate distribution of qualified staff among geographic and health system levels, low and underfunded salaries, lack of incentives (financial, training, personal development), and inadequate reimbursement of costs leading to poor morale and low productivity among staff. Towards the end of 2013, the quota for the health workforce was increased by 4 000 but most of these workers were deployed in cities and urban centers, thus resulting in little support for malaria services in remote areas [4].

Malaria elimination requires skillful staff in health facilities at all levels, however, the facilities that treat malaria currently are understaffed, which compromises the ability of the malaria program to detect and respond to outbreaks. The contrasting epidemiological dynamics between the northern and southern regions with an unequal human resource distribution within the vertical malaria program poses an even greater layer of complexity in being able to efficiently provide protection to the highest risk groups, including forest workers and soldiers [19].

### Laws and legal frameworks

Numerous laws exist to safeguard the health and welfare of workers [28, 29] including migrant or foreign labor, as well as conditions for the employer [30]. However, in the context of malaria, the worksite areas are generally remote and lack access to health, education, and basic services. Thus, implementation and enforcement remains a challenge, with affected workers likely to be illiterate, belong to an ethnic minority group, and live in the poorest households.

In 2009, the government agreed to allow the formation of Lao non-governmental organizations (NGOs). This decision was believed to encourage the creation of a stronger civil society [31] and to champion the rights of migrants, amongst others reasons. A more detailed review on how this has progressed since then is warranted but beyond the scope of this paper.

## Conclusions

Member countries committed to adopting the World Health Assembly Resolution WHA68.2 on the WHO Global Technical Strategy for Malaria 2016–2030 and the GMS elimination strategy 2015–2030 in May 2015. The Lao PDR’s 2015–2020 National Strategy for Malaria Control and Elimination outlines the strategies to progressively roll out the elimination of all species of malaria in selected provinces. Lao PDR’s prime minister has also signed the Declaration to Eliminate Malaria by 2030 in the Association of South East Asian Nations (ASEAN) Region at the 9th East Asia Summit. Although the challenges are numerous, the outlook is optimistic if efforts are made on a few critical fronts.

Firstly, bolder investment in social sector spending should be geared towards improving health service provision and utilization [4], ensuring equitable access to primary health care (including malaria) through efforts to achieve its universal health coverage targets. This should be extended to populations that are mobile or where accesses to health services are an obstacle either to physical access or to legal status of certain populations. In the case of malaria elimination, it is essential to reach all of the populations at risk, in particularly those in remote locations, as well as difficult to reach workplaces and migrant communities [3]. Integration of health services, decentralization, and restructuring should not translate to a diminishing quantity and quality of the workforce required to achieve malaria elimination targets. Raising government employment quotas, redistribution of tasks and health services, capacity-development activities and cross-training of the healthcare workforce [14], recruiting national staff with international experience, and recruiting professionals from the private sector [32] are proposed options to ensure a health workforce dedicated to the implementation and reporting of malaria activities [4]. Whilst acknowledging the challenges of maintaining this cadre of volunteers and workers over time, support from civil society organizations can create an enabling environment for training, supportive supervision, and service delivery.

Secondly, although the current population movement to forested areas and development projects is economically and financially driven and cannot be entirely stopped, in parallel with efforts to increase access through innovative strategies considering the mobility patterns and preferred access to health services of MMPs, enforcement of existing regulations should be accelerated to protect the workers and local populations living in these outbreak areas. In the short term, a multisector provincial action plan needs to be developed with the involvement of relevant agencies in agriculture, forestry, labor, energy and mining, as well as local community organizations, local authorities, and the military. A prerogative of the provincial governor should be to mandate all companies and project developers registered with the province to appoint focal point/s for health-related issues. A relook on agreements and in particular the possibility of channeling tax revenues collected from private companies on land use and forestry-related activities towards health-related activities, including malaria control and its elimination, should be explored. The Provincial Health Department, in turn, could ensure all health investment in the province is aligned with a provincial health integration plan. At a higher level, the MoH should create a subcommittee within the National Emerging Infectious Diseases Coordinating Office (NEIDCO) established under the prime minister’s office to oversee local government authorities to support policy and enforcement issues related to private sector project development in the provinces.

Thirdly, malaria elimination strategies will require cross-border cooperation with Lao PDR’s neighboring countries. Introduction of bilingual malaria patient cards that contain essential malaria information to be shown at any health care facility supported by SMS or mHealth platforms [24] will contribute to strengthened malaria surveillance, exchange of real-time essential data, and index case investigation across borders or in selected twin cities (district-district or district-township) where there is a malaria burden.

Fourthly, the engagement with the private sector would be essential, especially in the timely and effective implementation of health and environmental impact assessments, with private sector companies engaged in development and extractive industries [14]. This engagement should extend beyond Corporate Social Responsibility (CSR) capacities and facilitate dialogue between the health and private for-profit sectors on how to balance economic opportunities associated with domestic (local) health needs, services, and equity issues [33, 34]. Ensuring health care coverage of the informal sector is the most difficult challenge. The PPM initiative in Lao PDR for EDAT holds a lot of promise and should be reviewed to possibly expand its policy on engagement with the non-formal or unregistered drug outlets and vendors with suitable incentives for accreditation and licensing. This expansion would also create complimentary sentinel surveillance sites for the detection of malaria outbreaks that could be missed in the public sector health facilities.

Fifthly, the Lao malaria control program currently largely relies on LLINs and, in cases of outbreaks, indoor residual spray [2, 3]. Evidence shows, however, that limited levels of control are achieved with LLINs among highly mobile populations [35]. Existing and new interventions, which also target mosquitoes outside of human habitations, must be combined into integrated packages that control human exposure to mosquitoes at multiple points in the mobility pathway [36]. This may need innovative delivery mechanisms through social marketing approaches and the private sector rather than being fully reliant on the malaria vertical program [5]. The preference for conventional untreated nets (versus LLINs) and certain forms of mosquito repellents, especially in southern Lao PDR, also needs to be explored and appropriate strategies developed.

Sixthly, while acknowledging that timely access to malaria information and treatment, and availability of preventive and treatment services is of utmost importance to malaria control and elimination, some interventions need to be specifically tailored to the particular circumstances of these non-homogenous populations. These should factor in pathways of movement, seasonality and timing of departure, transit, and arrival of high‐risk groups including short‐ and long‐term forest workers, their accompanying families, MMPs, and the military. This needs to be done in collaboration with a broader involvement of civil society, local NGOs, and indeed by direct representation of people affected by malaria in in ensuring ‘MMP sensitive’ approaches and interventions are adopted depending on the mobility context and the acceptance of the population

## Additional file

Additional file 1: Multilingual abstracts in the five official working languages of the United Nations. (PDF 606 kb)

## Abbreviations

API: Annual parasite incidence
CMPE: Center for malariology, parasitology, and entomology
EDAT: Early diagnosis and treatment
GMS: Greater Mekong Subregion
ICT: Information and communications technology
Lao PDR: Lao People’s Democratic Republic
LLIH: Long-lasting insecticide-treated hammock
LLIN: Long-lasting insecticide-treated net
mHealth: Mobile health
MIS: Malaria information system
MMP: Mobile and migrant population
MoD: Ministry of defense
MoH: Ministry of health
NGO: Non-governmental organization
PPM: Public-private mix
SMS: Short messaging serviceTBA: traditional birth attendant
TES: Therapeutic efficacy study
VHV: Village health volunteer
WHO: World Health Organization
