# Supplementary material for: Malaria elimination in Lao PDR: the challenges associated with population mobility
Source: Infect Dis Poverty. 2017 Apr 25;6:81. doi: 10.1186/s40249-017-0283-5 (PMC5404311; doi:10.1186/s40249-017-0283-5)
Supplement: Additional file 1: — Multilingual abstracts in the five official working languages of the United Nations. (PDF 606 kb) [file 40249_2017_283_MOESM1_ESM.pdf]

Translation of the abstract into the five official working languages of the United Nations

التخلص من الملاريا في جمهورية لاو الديمقراطية الشعبية: التحديات المرتبطة بتنقل السكان

سينجتشان كونافونج، ديير جوبيناث، بواسي هونجفانتونج، تشانتالون كامكونج، أوداي سيتشانتونجتيب

ملخص

على الرغم من أن جمهورية لاو الديمقراطية الشعبية هي بلد غير ساحلي صغير نسبيا وبها أنماط هجرة داخلية وخارجية، إلا إنه لم تتم دراسة وضع الهجرة فيها بشكل مستفيض. هذا على الرغم من كل المحافظات الثماني عشر في البلاد بها نقاط تفتيش رسمية وغير رسمية على الحدود مع الدول المجاورة. شهدت الإصلاحات الاقتصادية في العقد الماضي زيادة تدريجية في تشجيع الاستثمار الأجنبي، وتوسعت المدن الرئيسية وشبكات النقل مما أتاح فرصا جديدة لكسب العيش والأنشطة الاقتصادية.

في العقد الماضي، كان هناك أيضا انخفاض كبير في حالات الملاريا المبلغ عنها في جمهورية لاو الديمقراطية الشعبية وفي حين أن هذا هو شرط هام للقضاء على الملاريا في البلاد، يشير تفشي الملاريا المبلغ عنها في السنوات الأربع الماضية إلى أن تنقل السكان، ولا سيما في الجنوب ، هو عامل مهم يعيق جهود المكافحة الحالية.

وينبغي أن يوجه الاستثمار بشكل أكثر جرأة للإنفاق على القطاع الاجتماعي من أجل تحسين تقديم الخدمات الصحية والاستفادة منها، وضمان المساواة في الحصول على الرعاية الصحية الأولية (بما في ذلك الملاريا) من خلال الجهود المبذولة لتحقيق أهداف التغطية الصحية الشاملة. ويجب أن يمتد هذا إلى السكان المتنقلين والمهاجرين. والحكومة المحلية تلعب دورا حاسما في دعم السياسات والتسهيلات المتعلقة بمشروعات القطاع الخاص للتنمية في المحافظات. والمبادرات عبر الحدود مع الدول المجاورة، وخاصة من حيث تبادل البيانات، والمراقبة، والاستجابة، أمر ضروري. وآليات إشراك القطاع الخاص، ولا سيما القطاع الخاص غير الرسمي، تحتاج إلى استكشافها في إطار اللوائح والقوانين المعمول بها. ويجب دمج الحلول القائمة والجديدة لمكافحة انتقال الملاريا عبر الهواء الطلق، لا سيما في الغابات، للفئات المعرضة للخطر بما في ذلك العمالة القصيرة والطويلة الأجل في الغابات وأسرهم، والسكان المتنقلين والمهاجرين، وكذلك الجيش في حزم متكاملة ذات آليات تنفيذ مبتكرة من خلال نهج التسويق الاجتماعي. يجب أن يحدث هذا في نقاط متعددة في مسار الحركة وإشراك القطاع الخاص بدلا من الاعتماد بشكل كامل على برنامج الملاريا الوطني ذي التوجه الرأسي.

بني المقال على مراجعة الأدبيات الموجودة من الملخصات والنصوص الكاملة، وتشمل المنشورات والأدبيات المحكمة باللغة الإنجليزية المأخوذة من PubMed والمؤلفات غير معلنة التي حُصل عليها من جوجل والباحث العلمي من جوجل. شمل الاستعراض أيضا دراسات حالة وتقارير القطاع، ووقائع المؤتمرات وتقارير البحوث والدراسات الوبائية والدراسات النوعية، وتقارير التعداد باللغتين اللاوية والإنجليزية. واستخدم واضعو البحث المصطلحات التالية: الملاريا والسكان المتنقلين، برامج مكافحة الملاريا والقضاء عليها، أداء النظام الصحي، تفشي الملاريا، جمهورية لاو الديمقراطية الشعبية. وشمل المواد المنشورة حتى يونيو 2015.

Translated from English version into Arabic by Mahmoud Sami, through

TRANSLATORS WITHOUT BORDERS

老挝疟疾消除过程中人口流动方面的相关挑战

Sengchanh Kounnavong, Deyer Gopinath, Bouasy Hongvanthong, Chanthalone Khamkong, and Odai Sichanthongthip

摘要：

尽管老挝是一个相对较小的、有人口流动的内陆国家，但其人口移徙状况研究不足。由于该国 18 个省同时拥有与邻国官方和非官方的边防检查站。过去十年的经济改革中，外国投资迅猛增加、主要城镇和交通网络极速扩张，给老挝经济活动和民生带来巨大的机遇。

过去十年，老挝报告的疟疾病例大幅减少，这是消除疟疾一个重要的先决条件。同时，过去 4 年的疫情报告显示，流动人口特别是在南部地区，是目前控制工作的一项重要挑战。
社会部门支出的大量投入应该针对改善卫生服务的提供和利用，保证公平获得初级卫生保健（包括疟疾）并通过努力实现全民健康覆盖的目标，这些红利同时也应该扩大到流动人口和移居人口中。在与私营部门项目开发有关的省份，当地政府在政策支持和执行方面起着至关重要的作用。与周边国家的跨境项目合作是至关重要的，特别是在数据共享、监测和响应方面。使私营部门参与其中，特别是非正式的私营部门的参与机制需要在现有法律法规的框架下进行探讨。现有的和新的针对户外疟疾传播干预措施，尤其是在森林环境中，对于包括短期和长期森林工人及其家庭、流动人口和移居人口以及军队等在内的高危人群，必须通过社会营销的方法以创新交付机制进行整合。而这应该发生在流动路径的多个节点并涉及私营部门，而不是完全依赖国家疟疾的垂直项目。
本文是对包括来自 Pubmed 已出版的经同行评议的英文文献，以及谷歌和谷歌学术的灰色文献等现有文献的摘要和全文进行了综述。本综述包含在以老挝文和英文发表的所有病例报道、部门报告、会议记录、研究报告、流行病学研究、定性研究和人口普查报告。检索词为：疟疾和流动人口、疟疾控制和消除项目、卫生系统性能、疟疾疫情暴发、老挝，检索截止日期为 2015 年 6 月。

Translated from English version into Chinese by Xin-Yu Feng, edited by Pin Yang

CHINESE CENTER FOR DISEASE CONTROL AND PREVENTION
NATIONAL INSTITUTE OF PARASITIC DISEASES

Éradication de la malaria au Lao RDP: Les enjeux associés à la mobilité de la population

Sengchanh Kounnavong, Deyer Gopinath, Bouasy Hongvanthong, Chanthalone Khamkong, et Odai Sichanthongthip

Résumé

Bien que la République Démocratique Populaire du Lao (Lao RDP) est un pays relativement petit, sans accès à la mer, avec des dispositions de migration interne et externe, la migration humaine du pays est mal étudiée. Et ceci malgré que les 18 provinces du pays aient des postes de contrôle frontaliers, officiels et non officiels, avec les pays voisins. Les réformes économiques de la dernière décennie ont mené à une augmentation graduelle de la promotion d'investissement étranger, et les villes principales et réseaux de transport se sont étendus ; offrant donc des nouvelles opportunités pour la subsistance et les activités économiques.
Durant la dernière décennie, une réduction importante des cas signalés de malaria a aussi été observé dans la RDP Lao et, tandis que c'est une condition préalable importante pour l'éradication de la malaria dans le pays, les épidémies de la malaria signalées lors des derniers 4 ans suggèrent que la mobilité de la population, particulièrement au sud, est un facteur important qui représente un défi aux efforts de contrôle courants.
Des investissements audacieux sur la dépense du secteur social devraient être orienter vers le développement de la provision et de l'utilisation du service de santé, assurant un accès équitable au soin de santé préliminaire (y compris de la malaria), à travers les efforts en vue d'atteindre les buts d'expansion de santé universels. Ceci devrait s'étendre vers les populations nomades et les migrants. Le gouvernement local joue un rôle critique de soutien des problèmes de politique et d'exécution liés au développement des projet du secteur privé dans les provinces. Les initiatives transfrontalières avec les pays voisins, surtout en termes de partage des données, surveillance et réponses, sont essentielles. Les mécanismes en vue d'engager le secteur privé, surtout le secteur privé informel, devraient être explorer dans le contexte des régulations et

lois existantes. Des interventions nouvelles et en cours pour la transmission externe de la malaria, particulièrement dans les milieux forestiers, pour des groupes à haut risque, comme des travailleurs de longue et courte durée et leurs familles, des populations nomades et migrante, et aussi l'armée, devraient être combinées en groupe intégré en vue de créer des mécanismes de livraison à l'aide des méthodes de marketing social. Ceci devrait se passer à des moments multiples le long de la voie de mobilité et impliquer le secteur privé, au lieu de ne faire référence qu'au programme vertical national de malaria.
Cet article basé sur l'évaluation de littérature existante, du résumé au texte complet, comprend la littérature de la langue anglaise publie et évaluée par des pairs, en provenance de Pub Med, et aussi des sources de littératures grises en provenance de Google et Google Scholar. Cette révision comprend aussi des rapports de cas, rapports sectoriels, actes de conférence, rapports de recherche, des études épidémiologiques, qualitative et des recensements dans les langues anglaise et du Laos. Les auteurs ont utilisé les termes de recherche : malaria et mobilité de la population, programme de contrôle de la malaria et éradication, performance du système de santé, épidémie de la malaria, RDP Lao ; et des articles publiés jusqu'en juin 2015.

Translated from English version into French by Ejila Makangu, through

TRANSLATORS
WITHOUT BORDERS

Ликвидация малярии в Лаосской НДР: трудности, связанные с мобильностью населения

Сенгчач Коуннавонг (Sengchanh Kounnavong), Дейер Гопинаф (Deyer Gopinath), Боуаси Хонгванфонг (Bouasy Hongvanthong), Чанфалоне Хамконг (Chanthalone Khamkong) и Одай Сичанфонгфип (Odai Sichanthongthip)

Аннотация

Лаосская Народно-Демократическая Республика (Лаосская НДР) – сравнительно небольшая, окружённая со всех сторон сушей страна. Тем не менее, в Лаосской НДР существует поток иммигрантов в страну и из неё, и ситуация с иммиграцией людей пока ещё плохо изучена, несмотря на то что все 18 провинций Лаосской НДР имеют общие официальные и неофициальные контрольно-пропускные пункты на границах с соседними странами. Экономические реформы, проведённые в последние 10 лет, привели к постепенному росту иностранных инвестиций. Главные города и транспортные сети продолжают развиваться, предлагая тем самым новые возможности для жизнеобеспечения и хозяйственной деятельности.
За прошедшее десятилетие в Лаосской НДР существенно снизилось количество известных случаев заболевания малярией. В то время как это является важным необходимым условием для ликвидации малярии, вспышки заболеваний, зарегистрированные за последние 4 года, указывают на то, что мобильность населения, особенно в южной части страны, сильно затрудняет принимаемые в настоящее время меры по борьбе с болезнью.
Увеличение капиталовложений в социальный сектор должно быть направлено на улучшение оказания медицинской помощи и использования ресурсов здравоохранения с обеспечением равного доступа к первичному медицинскому обслуживанию (включая лечение малярии) за счёт достижения всеобщего охвата услугами здравоохранения. Это также должно распространяться на мобильное население и иммигрантов. Роль местного правительства критически важна при поддержке постановлений и контроле над их соблюдением при внедрении

в провинциях частных проектов. Обязательно проведение трансграничных мероприятий с соседними странами, главным образом в отношении обмена данными, наблюдении и принятии ответных мер. Необходимо изучить в рамках существующих положений и законов механизмы привлечения частного сектора, в первую очередь неформального частного сектора. Следует объединить в комплексные пакеты с передовыми методами поставки с помощью социальных маркетинговых подходов уже имеющиеся и новые способы борьбы с переносчиками малярии в природных условиях, особенно в лесах, для групп высокого риска, включая рабочих, занятых на краткосрочных и длительных работах в лесу, а также членов их семей, мобильное население, иммигрантов и военнослужащих. Это необходимо сделать во многих местах на маршрутах передвижения населения и вовлекать частный сектор вместо того, чтобы полностью полагаться на национальную вертикальную программу борьбы с малярией.
Данная статья, основанная на обзоре существующей литературы от аннотаций до полных текстов, включает рецензированные публикации на английском языке из архива Pubmed и из «серых» источников, найденных с использованием Google и Google Scholar. В этот обзор также входят истории болезней, доклады по сектору, материалы конференций, отчёты об исследованиях, эпидемиологические изучения, качественный анализ и результаты переписи населения на лаосском и английском языках. Авторы пользовались при поиске ключевыми словами: малярия и мобильное население, программа по борьбе и ликвидации малярии, эффективность системы здравоохранения, вспышка малярии, Лаосская НДР; и включили статьи, опубликованные до июня 2015 г.

Translated from English version into Russian by Natalia Potashnik, through

TRANSLATORS
WITHOUT BORDERS

Eliminación de la malaria en la República Democrática Popular Lao: retos asociados a la movilidad de la población

Sengchanh Kounnavong, Deyer Gopinath, Bouasy Hongvanthong, Chanthalone Khamkong y Odai Sichanthongthip

Sinopsis
Aunque la República Democrática Popular Lao (RDP Lao) es un país sin costa relativamente pequeño con flujos tanto de inmigración como de emigración, se han realizado pocos estudios sobre la situación de sus migraciones humanas, a pesar de que las 18 provincias del país cuentan con puestos fronterizos oficiales y extraoficiales con los países de su entorno. Las reformas económicas realizadas durante la última década han supuesto un incremento gradual de la promoción de la inversión extranjera, y a medida que se han ido expandiendo las principales ciudades y redes de transporte han surgido nuevas oportunidades de formas de subsistencia y actividades económicas.
En los últimos diez años también ha habido una importante reducción en el número declarado de casos de malaria en la RDP Lao. Aunque esto supone un importante requisito previo para la eliminación de la malaria en el país, los brotes de malaria detectados en los últimos cuatro años parecen indicar que la movilidad de la población, especialmente en el sur, supone un importante impedimento a los actuales esfuerzos de control.
La mayor inversión en gastos sociales debería ir enfocada a una mejora de la provisión y uso de servicios sanitarios, al aseguramiento del acceso equitativo a la atención sanitaria primaria (incluyendo la malaria) mediante esfuerzos para

alcanzar objetivos de cobertura sanitaria universal. Esta cobertura debería abarcar también a las poblaciones en movimiento y migrantes. El gobierno local juega un papel crítico en el apoyo de cuestiones sobre política y aplicación relacionadas con el desarrollo de proyectos en el sector privado en las provincias. Las iniciativas transfronterizas con los países vecinos, especialmente relacionadas con la información compartida, vigilancia y respuesta, son esenciales. Se han de investigar mecanismos para involucrar al sector privado, especialmente el informal, dentro del marco de reglamentos y leyes actuales. Las intervenciones existentes y nuevas para la transmisión al aire libre de malaria, especialmente en entornos boscosos, para grupos de alto riesgo (incluyendo trabajadores forestales de corta y larga duración y sus familias, poblaciones en movimiento y migrantes, así como el ejército) se han de combinar en paquetes integrados con mecanismos innovadores de aplicación mediante enfoques de comercialización social. Esto se debería producir en diversos puntos en la ruta de movilidad, y debería incluir al sector privado en lugar de confiar solamente en el programa vertical de malaria del país.
Este artículo se basa en la revisión de literatura existente a partir de sinopsis y textos completos e incluye documentación en inglés publicada y revisada por pares obtenida a través de Pubmed así como fuentes de literatura gris a través de Google y Google Scholar. La revisión también incluye informes de casos, informes sectoriales, procedimientos de conferencias, informes de investigación, estudios epidemiológicos, estudios cualitativos e informes censales en inglés y en lao. Los autores emplearon los siguientes términos de búsqueda: malaria y poblaciones móviles, eliminación y programa de control de la malaria, rendimiento del sistema de salud, brote de malaria, RDP Lao, e incluyeron artículos publicados hasta junio de 2015.

Translated from English version into Spanish by Denis Smyth, through

TRANSLATORS
WITHOUT BORDERS
